# Living With HIV in Silence: Stigma, Disclosure and Barriers to Antiretroviral Therapy Among Women in Indonesia

**DOI:** 10.1155/arat/7327626

**Published:** 2026-11-04

**Authors:** Tintin Sukartini, Nursalam, Erna Dwi Wahyuni, Rio Ady Erwansyah, Achmad Masfi, Ronal Surya Aditya

**Affiliations:** ^1^ Universitas Airlangga Surabaya Indonesia unair.ac.id; ^2^ STIKes Hutama Abdi Husada Tulungagung Tulungagung Indonesia; ^3^ Universitas Negeri Malang Malang Indonesia um.ac.id; ^4^ Universitas Jember Jember Indonesia unej.ac.id

**Keywords:** antiretroviral agents, disclosure, family relations, HIV infections, medication adherence, qualitative research, social stigma

## Abstract

**Background:**

Although antiretroviral therapy (ART) has improved survival among people living with HIV, stigma and psychosocial barriers continue to hinder treatment adherence, particularly among women in collectivist and gendered sociocultural contexts. Women often experience HIV infection within intimate partnerships and may face compounded stigma that affects disclosure and engagement in care. However, limited qualitative research has explored how relational and family dynamics shape these experiences in Indonesia.

**Aim:**

To explore the lived experiences of women living with HIV in relation to stigma and barriers to ART adherence and to examine how relational and family dynamics influence treatment engagement.

**Methods:**

A qualitative descriptive study was conducted between February and July 2023 in Indonesia. Twenty‐two women living with HIV were recruited using purposive sampling. Data were collected through semistructured, in‐depth interviews and analysed using thematic analysis following Braun and Clarke’s framework. The study adhered to COREQ guidelines.

**Results:**

Five interconnected themes emerged: (1) relational pathways of HIV transmission, (2) internalised and enacted stigma, (3) negotiating disclosure, (4) physical and psychological barriers to ART and (5) family support as a buffering mechanism. HIV infection frequently occurred within intimate partnerships, reflecting relational vulnerability. Stigma influenced emotional well‐being, disclosure decisions and treatment engagement. Medication side effects, treatment fatigue and perceived wellness contributed to adherence challenges. Family support mitigated stigma‐related distress and facilitated sustained ART adherence.

**Conclusion:**

Women’s experiences of HIV are shaped by relational and sociocultural dynamics that influence stigma, disclosure and adherence. Gender‐sensitive, stigma‐informed and family‐inclusive nursing interventions are essential to strengthen long‐term treatment engagement among women living with HIV.

## 1. Introduction

Human immunodeficiency virus (HIV) remains a major global public health concern, with an estimated 39 million people living with HIV (PLHIV) worldwide, of whom women and girls account for a substantial and vulnerable proportion of new infections and overall prevalence [1–4]. Although the scale‐up of antiretroviral therapy (ART) has substantially reduced HIV‐related morbidity and mortality, sustained adherence remains essential to achieving viral suppression and preventing onward transmission [5–7]. However, ART adherence is not determined solely by biomedical access; social, psychological, and structural determinants continue to shape treatment engagement, particularly in low‐ and middle‐income countries [8].

Among these determinants, HIV‐related stigma has been consistently identified as a critical barrier to testing, disclosure, retention in care and treatment adherence [9, 10]. Stigma operates across multiple levels, including internalised shame, anticipated discrimination and enacted social exclusion, all of which adversely affect mental health and health‐seeking behaviours [11–13]. In Indonesia, women constitute a significant and growing proportion of PLHIV, with recent estimates indicating over 180,000 women aged 15 and over living with the virus [8]. For these women, HIV‐related stigma is not a monolithic experience but operates across multiple, interrelated dimensions: internalised (self‐directed shame and guilt), anticipated (fear of future discrimination), enacted (experienced acts of exclusion or prejudice) and structural (systemic barriers embedded in healthcare and social institutions) [14]. Stigma is further compounded by gender norms and moral judgement, particularly in contexts where HIV is associated with sexual misconduct [15–17]. These intersecting pressures may influence disclosure decisions, restrict social participation and ultimately undermine adherence to ART.

The sociocultural context of HIV in Indonesia presents a unique and complex landscape. Indonesia is defined by its national philosophy of Bhinneka Tunggal Ika (Unity in Diversity), encompassing hundreds of ethnic groups and diverse religious beliefs. Despite this vast cultural diversity, unifying sociocultural threads—such as strong collectivism, patriarchal family structures and deep religious moralisation—profoundly shape health behaviours and stigma across the archipelago. In this context, adherence to ART among women is uniquely complicated by gender‐specific physical side effects, the psychological burden of chronic illness management and the compounding effect of HIV‐related stigma, which can severely deter consistent treatment engagement and exacerbate health disparities. In this context, it is crucial to distinguish between, yet understand the intersection of, HIV‐related stigma and broader sociocultural stigma. HIV‐related stigma is primarily driven by moral judgements associating the virus with perceived sexual misconduct or drug use. Conversely, sociocultural stigma in Indonesia is deeply rooted in the fear of bringing *aib* (family disgrace) and *malu* (profound shame/loss of face) to the collective family unit, disrupting communal harmony (*rukun*). For women, whose social value is closely tied to modesty and marital loyalty, these two stigmas intersect severely: An HIV diagnosis is frequently interpreted by the community not merely as a health issue, but as a confirmation of moral failure and a sociocultural transgression. While quantitative research has established associations between stigma and adherence, fewer qualitative studies have explored how these intersecting stigmas are experienced by women within this specific cultural framework of unity in diversity.

In many Asian and collectivist settings, including Indonesia, women’s vulnerability to HIV infection is frequently embedded within marital and relational dynamics. Studies have shown that women often acquire HIV from intimate partners and may have limited power to negotiate condom use due to economic dependence and sociocultural expectations of loyalty [18–20]. Furthermore, in communities where family reputation and religious participation are central to identity, disclosure of HIV status may carry significant social consequences. While quantitative research has established associations between stigma, social support and adherence [21, 22], fewer qualitative studies have explored how these processes are experienced and negotiated by women within specific cultural contexts.

Understanding how relational power dynamics, sociocultural norms (such as the fear of aib and malu) and structural barriers intersect to influence ART adherence is essential. This understanding is critical for developing contextually responsive nursing interventions, as nurses are often the primary point of contact in HIV care and are uniquely positioned to deliver holistic, family‐centred support that addresses these multifaceted barriers. However, limited research has examined these processes comprehensively (encompassing physical, psychological and sociocultural dimensions) among women living with HIV in Indonesia. While Western models of HIV stigma and social support predominantly focus on individualised moral judgement and peer‐led support networks, this study explores how HIV stigma intersects with collective sociocultural constructs (aib and malu) in a highly diverse, collectivist society. Specifically, Western frameworks often conceptualise stigma as an individual psychological burden, whereas in the Indonesian context, stigma operates as a structural threat to communal harmony (rukun) and family honour. An HIV diagnosis for a woman is not merely a personal health event, but a sociocultural transgression that brings aib (disgrace) to the collective family unit. Understanding this intersection is critical for designing culturally congruent interventions not only in Indonesia but across similar collectivist settings in the Global South, where familial and communal dynamics fundamentally dictate health behaviours and treatment cascades. Despite growing recognition that HIV‐related stigma and social support influence treatment engagement, an important conceptual question remains insufficiently resolved: Under what relational conditions does family support protect women from stigma‐related barriers, and when might family involvement introduce additional risks to autonomy, confidentiality or disclosure safety? Treating family support as uniformly beneficial may obscure meaningful differences in the quality and safety of family relationships. Emotional acceptance, practical assistance and medication reminders may facilitate engagement in care, whereas coercive disclosure, blame or controlling responses may intensify vulnerability. These possibilities are particularly relevant when women must negotiate treatment within intimate partnerships and family networks in which HIV status may carry moral and social consequences. Accordingly, this study approaches family support not simply as an individual resource, but as a relational process situated within broader sociocultural conditions. By examining how women describe stigma, disclosure, treatment barriers and family responses together, the study provides a contextually grounded basis for developing a provisional relational account of HIV care engagement. This perspective is intended to generate testable propositions for subsequent research rather than presume causal mechanisms that cannot be established through a qualitative descriptive design. Therefore, this study aimed to explore the lived experiences of women living with HIV in relation to stigma and barriers to ART adherence and to examine how relational and family dynamics shape treatment engagement. By foregrounding women’s narratives within their sociocultural environment, this study contributes to the development of gender‐sensitive, stigma‐informed and family‐inclusive nursing approaches to HIV care.

## 2. Methods

### 2.1. Study Design

This study employed a qualitative descriptive design to explore the lived experiences of women living with HIV in relation to stigma and barriers to ART. A qualitative descriptive approach was selected as it is best suited for providing a comprehensive, straight description of participants’ experiences, allowing for the identification of patterns and themes without the deep philosophical abstraction required in pure phenomenology. The study was informed by constructivist principles, recognising that participants’ experiences are shaped by their social, relational and cultural contexts. Reporting of the study followed the Consolidated Criteria for Reporting Qualitative Research (COREQ) guidelines. Thematic analysis following Braun and Clarke’s six‐step framework was used as the primary analytic strategy.

### 2.2. Setting

The study was conducted in collaboration with the AIDS Prevention Commission (Komisi Penanggulangan AIDS, KPA) in Tulungagung Regency, East Java, Indonesia. Tulungagung is a semiurban area with established HIV care services delivered through community health centres (Primary Health Centres) and referral hospitals. The KPA provides counselling, coordination of care and community‐based support for PLHIV. Crucially, the KPA plays a pivotal role in this setting by acting as a bridge between clinical services and community‐based support, making it an ideal setting to capture the relational and structural dynamics of HIV care in a semiurban Indonesian context. While Tulungagung is predominantly Muslim and semiurban and thus does not capture the entirety of Indonesia’s vast cultural diversity, it represents a highly relevant sociocultural microcosm of Java. The core collectivist values observed here (e.g., family honour, aib, malu) are deeply resonant across many Indonesian contexts, although we explicitly caution against overgeneralisation, as detailed in the Limitations section.

### 2.3. Participants and Sampling

Participants were women living with HIV who were registered with the local KPA and aged 18 years or older. Inclusion criteria were as follows: (1) confirmed diagnosis of HIV, (2) currently receiving or having previously received ART and (3) ability to communicate experiences verbally. Women with severe cognitive impairment or inability to participate in an interview were excluded. Purposive sampling was used to recruit participants who could provide rich and diverse descriptions of stigma and treatment experiences, ensuring maximum variation in age, educational background and duration of HIV infection to capture a comprehensive range of lived experiences. KPA coordinators informed eligible participants about the study. Interested individuals were contacted directly by the research team and provided with detailed written and verbal explanations of the study. Recruitment continued until data saturation was reached. Saturation was defined as the point at which no new codes, categories or themes emerged during iterative analysis of subsequent interviews. All participants who provided consent completed the interviews, and no participants withdrew from the study. In this study, thematic saturation was initially achieved at the 20th interview, with two additional interviews conducted to confirm that no new codes, categories or conceptual insights were emerging, bringing the final sample to 22 participants.

### 2.4. Research Team and Reflexivity

Interviews were conducted by two female researchers (TS and RSA) with backgrounds in mental health nursing and prior training in qualitative research. At the time of the study, both researchers were nursing lecturers and researchers involved in HIV‐related care and community health programs. No prior personal relationship existed between researchers and participants before recruitment. Before the interviews began, participants were informed about the researchers’ roles, the objectives of the study and the purpose of the interviews. To minimise bias, researchers engaged in bracketing throughout the study. Given the researchers’ professional backgrounds, we critically examined the inherent power dynamics and positionality within the research process. Participants might have perceived the researchers as healthcare authorities, potentially influencing their willingness to disclose nonadherence or sensitive stigma experiences. To mitigate this, researchers explicitly clarified their role as independent researchers rather than treating clinicians during the consent process. Furthermore, the use of the local language (Bahasa Indonesia) and culturally congruent communication styles was employed to minimise hierarchical distance, foster rapport and create a safe, egalitarian space for sharing vulnerable narratives. Reflexive journals were maintained continuously to document assumptions, emotional responses and analytic decisions during data collection and analysis. To ensure analytical rigour, the research team held regular coordination meetings and structured peer debriefing sessions to discuss emerging themes, challenge individual interpretive biases and reach consensus on coding decisions, thereby ensuring that clinical biases did not overshadow the participants’ authentic lived experiences.

### 2.5. Data Collection

Data were collected between February and July 2023. Semistructured, in‐depth interviews were conducted in private and comfortable locations chosen by participants, including KPA offices and community health facilities. Each interview lasted approximately 30–60 min. Repeat interviews were not conducted. No nonparticipants were present during the interviews. Prior to data collection, the interview guide with open‐ended questions was reviewed and validated by a panel of experts in qualitative research and HIV clinical care to ensure content validity, cultural appropriateness and sensitivity to the participants′ vulnerabilities. The validated guide was used to explore participants’ experiences related to the following:

1. HIV diagnosis and transmission
2. Experiences of stigma and discrimination
3. Disclosure decisions
4. Barriers to ART adherence
5. Family and community support

Probing questions were used to clarify responses and encourage deeper reflection. Interviews were audio‐recorded with participants’ consent. Field notes were taken to document nonverbal cues and contextual observations. Interviews were conducted in Bahasa Indonesia. Audio recordings were transcribed verbatim in the original language. Transcripts were translated into English for publication purposes. To ensure translation accuracy and conceptual equivalence, a forward‐backward translation method was employed. Bilingual members of the research team independently translated the transcripts, and discrepancies in culturally nuanced terms (such as aib or malu) were resolved through consensus to preserve the original emotional and cultural context. Transcripts were not returned to participants for comment or correction.

### 2.6. Data Analysis

Data were analysed using a six‐phase thematic analysis process informed by Braun and Clarke’s reflexive approach. The researchers first familiarised themselves with the transcripts, generated initial codes, developed candidate themes, reviewed and refined the themes against the dataset, defined and named the themes and produced the final analytical account. Coding was conducted manually, and members of the research team discussed interpretations and differences in coding during regular analytic meetings to reach a consensus understanding of the data. Reflexive journals and an audit trail were maintained to document assumptions, interpretive decisions and theme development. The analysis aimed to produce a coherent interpretation grounded in participants′ accounts while acknowledging the researchers′ professional backgrounds and the interpretive nature of qualitative analysis. A detailed methodological audit trail, including the de‐identified thematic coding framework and the completed COREQ checklist, is provided in Supporting File 1 to ensure full analytical transparency.

### 2.7. Trustworthiness

Trustworthiness was ensured through strategies addressing credibility, dependability, confirmability and transferability. Credibility was enhanced through prolonged engagement with participants, peer debriefing among the research team, and iterative comparison of data and emerging themes. Dependability was supported through maintenance of an audit trail documenting methodological decisions and analytic steps. Confirmability was strengthened through reflexive journaling and team discussions to minimise researcher bias. Transferability was facilitated by providing detailed descriptions of the study setting and participant characteristics. Member checking was not formally conducted; however, analytic interpretations were reviewed collaboratively within the research team to ensure consistency and alignment with participants’ narratives. Multiple researchers were involved in coding and theme development to enhance analytic rigour and reduce individual interpretive bias. Trustworthiness was ensured through strategies addressing credibility, dependability, confirmability and transferability. Credibility was enhanced through prolonged engagement with participants, peer debriefing among the research team and iterative comparison of data and emerging themes. Dependability was supported through the maintenance of a comprehensive audit trail, and confirmability was strengthened through reflexive journaling. The detailed methodological audit trail and the completed COREQ 32‐item checklist are explicitly provided in Supporting File 1. Transferability was facilitated by providing detailed descriptions of the study setting and participant characteristics.

### 2.8. Ethical Considerations

Ethical approval was obtained from the Health Research Ethics Commission of the Faculty of Nursing and Health, Muhammadiyah University Semarang (No. 103/KE/07/2023). Participants received written and verbal information about the study’s purpose, procedures, confidentiality and voluntary nature of participation. Written informed consent was obtained prior to interviews. Participants were informed that they could withdraw at any time without consequences. To ensure confidentiality, pseudonyms and participant codes (e.g., P1, P2) were used in transcripts and reporting. Audio recordings and transcripts were stored securely and accessible only to the research team. Written informed consent was obtained from all participants prior to data collection.

## 3. Results

This study explored the experiences of women living with HIV in relation to stigma and barriers to ART. Analysis of in‐depth interviews identified patterns across participants’ narratives while also capturing variations in individual experiences.

### 3.1. Participant Characteristics

Twenty‐two women living with HIV participated in this study. Participant characteristics are presented in Table 1. Participants’ ages ranged from 35 to 56 years (mean 43 years). Most were married (*n* = 15), five were widowed, one was divorced and one was single. Educational levels ranged from elementary (*n* = 4) to senior high school (*n* = 9). Ten participants were housewives, and 12 were self‐employed. The majority identified as Muslim (*n* = 21) (Table 1). Data saturation was achieved after the 20th interview, as no new themes or categories emerged.

**TABLE 1:** Participant characteristics (*n* = 22).

Characteristic	*n*	%
*Age (years)*		
35–44	14	63.6
45–56	8	36.4

*Marital status*		
Married	15	68.2
Widowed	5	22.7
Divorced	1	4.5
Single	1	4.5

*Education level*		
Elementary school	4	18.2
Junior high school	9	40.9
Senior high school	9	40.9

*Occupation*		
Housewife	10	45.5
Self‐employed	12	54.5

*Religion*		
Islam	21	95.5
Christian	1	4.5

Five interconnected themes emerged from the analysis (Table 2). Although each theme represents a distinct aspect of participants’ experiences, they were closely related and reflected a process beginning with infection within relational contexts, followed by stigma experiences, disclosure negotiation, treatment challenges and the role of family support.

**TABLE 2:** Themes and subthemes.

Theme	Subthemes
Relational pathways of transmission	Spousal transmission; pregnancy‐related diagnosis; needle sharing
Internalised and enacted stigma	Shame and self‐isolation; community discrimination
Negotiating disclosure	Selective trust; fear of moral judgement
Barriers to ART	Side effects; treatment fatigue; perceived wellness
Family support	Emotional reassurance; practical assistance; motivational reinforcement

#### 3.1.1. Theme 1: Relational Pathways of HIV Transmission

Most participants reported acquiring HIV from their husbands. Infection frequently occurred within marriage, often discovered after a partner’s prolonged illness. Several women expressed feelings of betrayal, yet also resignation due to economic dependence.

*‘I found out I was infected with HIV after my husband had been sick for almost three months’. (P5)*

Women described limited ability to negotiate condom use, particularly when financially dependent. A few participants who remarried reported inconsistent condom use despite awareness of risk.

Diagnosis during pregnancy was reported by several participants, who described shock and fear when learning their status during routine antenatal care.

*‘I was six months pregnant when I found out. I cried because I was thinking about my baby’. (P17)*

One participant described infection through needle sharing in adolescence, reflecting a different vulnerability pathway.

Overall, transmission was largely embedded within intimate relationships, indicating that infection frequently occurred within intimate relational contexts.

#### 3.1.2. Theme 2: Internalised and Enacted Stigma

Experiences of stigma were pervasive across participants and shaped emotional, social and treatment‐related responses and manifested as both internalised shame and enacted discrimination.

##### 3.1.2.1. Shame and Self‐Isolation

Most women described intense emotional reactions following diagnosis, including shame, fear and withdrawal.

*‘I locked myself in my room for four days. I felt dirty and ashamed’. (P4)*

Several participants concealed their status for years, fearing moral judgement and social exclusion. Women living in close‐knit communities described heightened anxiety about being labelled as immoral.

*‘Only my husband knows. If others know, they will think I am a bad woman’. (P11)*

##### 3.1.2.2. Community Discrimination

Many participants experienced discriminatory behaviours within family and community settings. Examples included the separation of utensils and exclusion from communal religious activities.

*‘My parents made a separate bathroom for me’. (P9)*

*‘I was told to stop attending Yasinan because they were afraid’. (P16)*

Given the central role of communal and religious gatherings in daily life, exclusion carried a significant emotional impact. Misconceptions about transmission persisted within the community.

#### 3.1.3. Theme 3: Negotiating Disclosure

Disclosure was not a single event but an ongoing negotiation. Most participants selectively disclosed their status to one trusted person, often a sibling or spouse.

*‘The first person I told was my older sister. I needed someone to understand’. (P1)*

For some, supportive responses strengthened emotional resilience and treatment commitment. Others delayed disclosure due to the fear of rejection or being blamed.

Widowed participants described additional challenges, as disclosure could influence remarriage prospects.

#### 3.1.4. Theme 4: Physical and Psychological Barriers to ART

Participants described both bodily discomfort and emotional fatigue as barriers to adherence, highlighting the profound physical and psychological toll of lifelong treatment.

##### 3.1.4.1. Side Effects

Most participants reported dizziness, nausea, tremors or skin reactions during early treatment phases.

*‘I felt dizzy and nauseous after taking the medicine’. (P2)*

Women with lower educational backgrounds appeared more anxious about side effects and were more likely to delay treatment initiation. Participants with lower educational backgrounds described greater uncertainty regarding medication side effects, whereas those with longer treatment experience reported better adaptation over time.

##### 3.1.4.2. Treatment Fatigue and Perceived Wellness

Beyond physical symptoms, participants articulated a deep emotional exhaustion associated with the chronicity of their condition. Several participants described boredom and emotional exhaustion associated with lifelong therapy.

*‘I feel tired of taking ARVs, but I remember my children who still need me’. (P6)*

*‘Because I feel fine, sometimes I think I don’t need to take the medicine every day’. (P8)*

Some participants perceived themselves as healthy and questioned the necessity of continuous medication, particularly when symptoms were absent. This reflects a cognitive dissonance where the absence of symptoms diminishes the perceived necessity of continuous medication, complicating long‐term adherence.

#### 3.1.5. Theme 5: Family Support as a Buffering Mechanism

Family support emerged as a critical protective factor mitigating stigma and strengthening adherence. Rather than functioning merely as a logistical aid, family support acted as an active, relational buffer against the psychological distress of stigma. Most participants who reported consistent treatment also described strong family involvement.

Support included medication reminders, accompaniment to healthcare services, emotional reassurance and profound expressions of acceptance.

*‘My sister always reminds me to take medicine’. (P8)*

*‘My husband accompanies me to the health center’. (P19)*

Participants who experienced acceptance described feeling more confident and less isolated. These acts of accompaniment and reminders were interpreted by the women not just as practical help, but as tangible validations of their worth and unconditional acceptance, directly countering the enacted stigma experienced in the broader community. In contrast, limited family support was associated with emotional distress and inconsistent adherence.

### 3.2. Integrative Model of Findings

Figure 1 illustrates the dynamic relationship among relational transmission, stigma experiences, disclosure processes, treatment barriers and the buffering role of family support. HIV infection, primarily occurring within intimate relationships, was followed by experiences of stigma that shaped psychological distress and disclosure decisions. These processes influenced barriers to ART adherence. Family support appeared to mitigate stigma‐related distress and was associated with more consistent treatment adherence.

**FIGURE 1:**
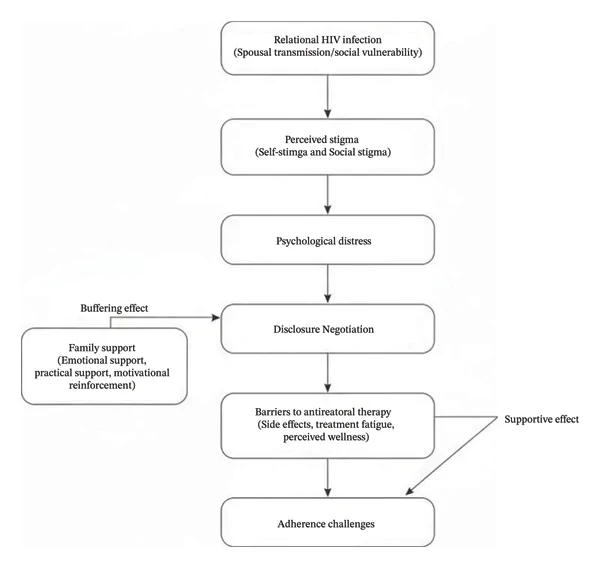
The Relational Buffering Model of HIV Care in collectivist settings. This model illustrates the dynamic pathway in which HIV infection, primarily occurring within intimate relationships, leads to experiences of intersecting stigma (moral and sociocultural). These experiences shape psychological distress and ongoing disclosure negotiations, which in turn influence physical and psychological barriers to ART adherence. Crucially, the model positions family support not merely as a logistical aid, but as the primary structural buffer that intercepts the pathway between sociocultural stigma (aib/malu) and treatment disengagement, thereby fostering resilience and sustained care engagement.

Overall, the findings demonstrate a relational and contextual pattern in which HIV infection occurred primarily within intimate partnerships, followed by pervasive stigma that influenced emotional responses, disclosure decisions and treatment adherence. Barriers to ART were shaped by both physical and psychological factors, while family support emerged as a consistent protective element across narratives. These interconnected findings provide insights into how social and relational dynamics shape the lived experiences of women with HIV and form the basis for further interpretation in the Discussion section.

## 4. Discussion

This study examined the lived experiences of women living with HIV in Indonesia, focusing on stigma, disclosure and barriers to ART. The findings reveal an interconnected process in which infection occurring primarily within intimate partnerships is followed by stigma experiences that shape disclosure decisions and treatment adherence. Within this process, family support emerges as a stabilising and protective influence. Together, these findings illuminate the relational and sociocultural dimensions of HIV care engagement among women.

A central finding of this study is that most participants acquired HIV within marriage. This pattern aligns with global evidence demonstrating that women in many low‐ and middle‐income countries are disproportionately infected through spousal transmission rather than individual high‐risk behaviours [21, 22]. Such patterns reflect broader gendered power dynamics, including limited negotiation capacity regarding condom use and economic dependence within marital relationships [23–25]. Importantly, the present findings extend this literature by illustrating how relational trust, marital loyalty and financial vulnerability intersect to constrain women’s agency. In this context, infection cannot be understood solely as an outcome of individual decision‐making, but rather as embedded within structural inequalities (e.g., systemic economic dependence, limited access to health literacy and reproductive resources, and institutional barriers to confidential HIV testing) and relational inequalities (e.g., gendered power imbalances within marriages that restrict a woman’s agency to negotiate safe sex, disclose her status or seek care without spousal permission). The women’s narratives reveal a profound humanistic tragedy: Their vulnerability is inextricably linked to the very intimate partnerships that are culturally expected to provide them with safety and security. These findings underscore the urgent need for gender‐sensitive and couple‐focused prevention strategies within nursing and community health practice.

Beyond transmission pathways, stigma emerged as a pervasive and multidimensional experience shaping participants′ interconnected social and relational spheres (including family, community and religious groups), each with its own distinct norms and expectations regarding HIV. Consistent with prior research, stigma manifested both internally, through shame and self‐withdrawal, and externally, through discrimination and social exclusion [26–28]. Internalised stigma has been linked to depression, reduced self‐efficacy and diminished treatment adherence [29–32], patterns strongly echoed in recent qualitative studies of women living with HIV in Indonesia, where internalised shame is frequently compounded by moral self‐judgement rooted in religious teachings [29–31, 33]. However, in the present study, stigma was also deeply intertwined with communal and religious life. In collectivist contexts such as Indonesia, social belonging and participation in religious activities (e.g., Yasinan, communal prayers) represent core components of a woman’s identity and moral standing. Exclusion from such spaces therefore amplifies psychological distress beyond individual shame, constituting a form of spiritual and social alienation that is particularly devastating in a society where religious participation is synonymous with moral legitimacy. This finding highlights that stigma operates not only at the psychological level but also within cultural and social structures—where patriarchal norms, religious moralisation and communal expectations of female modesty intersect to produce a uniquely gendered burden. Consequently, stigma‐reduction interventions must extend beyond individual counselling to encompass community‐level engagement, religious leader partnerships and culturally grounded psychoeducation that addresses the moral narratives underpinning discrimination.

To clarify the mechanisms of this marginalisation, it is essential to disentangle and examine the intersection of HIV‐related stigma and sociocultural stigma in the Indonesian context. HIV‐related stigma operates through moral labelling, where the virus is inextricably linked to promiscuity or immoral behaviour. However, sociocultural stigma operates through the lens of communal honour and social hierarchy. In a collectivist society, the individual is viewed as a representative of the family. Therefore, the enacted stigma experienced by participants—such as being excluded from communal religious gatherings (*Yasinan*) or subjected to physical segregation (e.g., separate bathrooms)—is not solely driven by a fear of viral transmission, but by a sociocultural imperative to distance the community from the *aib* (disgrace) associated with the woman. For women living with HIV, these stigmas do not operate in isolation; they intersect to create a compounded burden. The HIV‐related stigma attacks their moral identity as women, while the sociocultural stigma isolates them from the communal and spiritual belonging that is central to Indonesian daily life. This intersection explains why the psychological distress reported by participants extended far beyond individual shame to a profound sense of social and spiritual alienation.

Within the context of stigma, disclosure emerged as a strategic and ongoing negotiation rather than a singular event. Participants described selectively disclosing their HIV status to trusted individuals while weighing the possibility of rejection against the need for emotional and practical support [29–31]. This pattern is consistent with the view that disclosure decisions depend partly on anticipated responses and the perceived safety of interpersonal relationships. In the present study, the accounts indicate that fear of judgement and social exclusion shaped disclosure decisions and could limit access to informal support. However, the findings should not be interpreted as establishing that nondisclosure directly caused missed clinic appointments, concealment of medication or ART discontinuation, as these specific behaviours were not uniformly documented in the interview data [33–35]. Clinically, the findings support confidential, nondirective disclosure counselling that helps women assess potential benefits and risks without treating disclosure as a prerequisite for care. Nurses should respect each woman’s preferences and prioritise safety, autonomy and continuity of treatment.

In addition to stigma‐related influences, participants described medication side effects, treatment fatigue and perceived wellness as barriers to adherence. ART‐related adverse effects remain a recognised challenge in long‐term HIV management [36–38]. However, the present findings demonstrate that physical symptoms alone do not fully explain adherence difficulties. Rather, uncertainty, limited health literacy and emotional exhaustion compound these barriers. The perception of feeling physically well further complicated adherence behaviours, reflecting patterns documented in chronic disease management where asymptomatic periods reduce perceived necessity for medication [14, 39, 40]. These findings suggest that adherence support should move beyond symptom management to include continuous education, motivational reinforcement and mental health integration.

The findings support a relational interpretation of HIV care engagement in which the meaning and potential value of family support must be understood alongside the stigma and disclosure risks experienced by women living with HIV. We therefore propose the Relational Buffering Model of HIV Care Engagement as a provisional conceptual framework derived from the interconnected themes identified in this study [41–43]. The model suggests that family support may help women navigate stigma‐related distress through emotional acceptance, practical assistance and reinforcement of treatment routines. However, the potential buffering role of family support should not be assumed to be universal or independent of relational context. Its protective value may depend on whether support is experienced as voluntary, trustworthy, confidential and respectful of women’s autonomy. Conversely, family responses involving blame, coercion or unwanted disclosure could plausibly intensify distress and complicate treatment engagement, although these adverse pathways require further empirical examination. The present study establishes the relevance of relational and family processes in participants′ accounts; it does not establish statistical mediation, moderation or causal effects on ART adherence. The model therefore serves as a theory‐generating contribution that identifies relationships and mechanisms for testing in future multisite and longitudinal research. The proposed model generates three propositions for future investigation. First, perceived HIV‐related stigma may be associated with treatment disengagement partly through emotional distress and difficulties in negotiating disclosure. Second, supportive family relationships may weaken the association between stigma‐related distress and treatment disengagement when support preserves confidentiality and personal autonomy. Third, the relationship between family involvement and care engagement may vary according to the quality of the relationship, the woman’s disclosure preferences and the availability of confidential alternatives to family‐based support. These propositions require empirical testing and should not be interpreted as causal conclusions from the present qualitative study.

In this study, participants’ accounts suggest that concerns about family reputation, shame and community judgement were relevant to how some women experienced HIV‐related stigma and negotiated disclosure. Concepts such as aib and malu may help contextualise these accounts, but their meanings and implications should not be assumed to be uniform across Indonesia. The findings are situated in one semiurban setting in East Java and therefore do not establish that family‐based support is universally preferred or more effective than peer‐based or individual support. Rather, they highlight the importance of understanding how locally meaningful social expectations interact with each woman’s relationships, disclosure preferences and access to confidential care. Future comparative research should examine how these processes vary across regions and social contexts, including among women who experience family conflict or prefer support outside the family.

The cultural interpretation of family support requires an important qualification. Although concepts such as aib and malu helped contextualise participants’ accounts, this study does not establish that these constructs operate uniformly across Indonesia or that family‐centred approaches are inherently superior to peer‐based or individualised support. Indonesia’s cultural, religious and regional diversity makes such generalisation inappropriate. Family relationships may provide acceptance and practical assistance, but they may also involve conflict, unequal power or concerns about confidentiality. Similarly, peer support and community‐based services may offer valuable alternatives for women who cannot safely disclose their HIV status within their families. The practical implication is therefore not to prioritise family involvement universally, but to provide relationally responsive care in which women can choose the form of support that best meets their needs and circumstances. This qualification strengthens the proposed model by positioning relational safety, rather than family membership alone, as a potentially important condition of supportive care. Whether relational safety explains variation in the association between stigma and treatment engagement remains an empirical question for future research.

The findings have implications for nursing practice at individual, relational and service‐delivery levels. At the individual level, nurses should provide nonjudgemental ART counselling that addresses treatment fatigue, concerns about adverse effects and misconceptions arising when women feel physically well. At the relational level, nurses may offer voluntary family‐inclusive counselling when requested by the woman and when involvement is considered safe. Such counselling should focus on supportive communication, practical assistance, confidentiality and respect for the woman’s treatment decisions. Disclosure should remain a supported choice rather than a prerequisite for receiving care. At the service‐delivery level, HIV programmes may incorporate brief relational‐safety assessments into routine consultations to identify concerns about coercion, unwanted disclosure, family conflict or barriers to confidential access. Women who do not wish to involve relatives should retain access to individual counselling, peer support and other appropriate services. These recommendations represent practice implications derived from participants′ accounts, not interventions whose effectiveness has been demonstrated by this study. Future implementation research should evaluate feasibility, acceptability, fidelity, resource requirements, unintended harms and sustainability within routine nursing services. Patient‐centred outcomes should include perceived safety, autonomy, continuity of care and treatment engagement, with objective clinical indicators considered where ethically and practically feasible. The sustainability of a future family‐inclusive intervention should be assessed beyond initial acceptability or short‐term participation. Relevant implementation outcomes include continued delivery after initial research support ends, staff capacity to maintain the intervention, integration into existing clinic workflows, affordability, equitable access for women with different disclosure circumstances and continued protection of confidentiality. Evaluation should also examine whether the intervention remains appropriate across different healthcare settings and whether adaptations preserve its core principles of voluntariness, relational safety and women’s autonomy. These outcomes would help distinguish an intervention that is initially feasible from one that can be maintained responsibly within routine HIV care.

## 5. Strengths, Limitations and Future Research

This study provides an in‐depth account of how women living with HIV describe the interconnected experiences of relational vulnerability, stigma, disclosure, treatment barriers and family support within a semiurban Indonesian setting. The use of in‐depth interviews, reflexive documentation, collaborative analysis and contextual description supports a nuanced interpretation of participants’ accounts. An additional contribution is the development of a provisional Relational Buffering Model of HIV Care Engagement that translates the thematic findings into questions suitable for subsequent empirical investigation.

Several limitations should be acknowledged. First, participants were recruited through a single regional network in Tulungagung, East Java. Their experiences cannot be assumed to represent women living with HIV across Indonesia, where cultural practices, family structures, healthcare access and community responses may differ. Second, the findings rely on participants’ retrospective self‐reports and may be influenced by recall, social desirability and the sensitivity of discussing HIV status with researchers who have healthcare backgrounds. Third, the study did not independently verify medication‐taking behaviour, clinic attendance, viral suppression or other clinical outcomes. Consequently, references to adherence reflect participants’ accounts and perceived barriers rather than objectively measured treatment outcomes. Fourth, the qualitative descriptive design does not establish causal pathways or determine whether family support statistically moderates or mediates the relationship between stigma and treatment engagement. Fifth, the absence of formal participant transcript review limits the extent to which participants directly confirmed the researchers′ final interpretations. Finally, although the analysis was informed by reflexivity and collaborative review, the influence of researchers′ professional positions and interpretive assumptions cannot be eliminated completely.

Future research should proceed through a staged programme. Initial multisite qualitative studies should examine the variation in relational safety, disclosure preferences and family responses across different Indonesian settings, including experiences that challenge the proposed model. Subsequent quantitative studies should assess the measurement properties of relevant constructs and test associations between stigma, relational safety, family support and treatment engagement. Longitudinal designs would help establish temporal ordering, while carefully designed intervention studies could evaluate whether voluntary, safety‐informed family‐inclusive nursing approaches improve patient‐centred outcomes without increasing unwanted disclosure or relational harm. Implementation research should then examine feasibility, acceptability, fidelity, costs, equity and maintenance within routine HIV services. Cross‐cultural comparisons may determine which aspects of the model are context‐specific and which may be transferable to other settings. This programme would enable the proposed model to evolve from a qualitative, theory‐generating framework into an empirically examined and contextually adaptable approach to HIV care. Ultimately, this staged research agenda aims to transition the proposed Relational Buffering Model from a qualitative, theory‐generating framework into an empirically examined approach. Future implementation research must evaluate whether relationally responsive interventions can be sustained within routine healthcare services, ensuring they remain safe, acceptable and protective of women’s autonomy and confidentiality over the long term.

## 6. Conclusion

This study illustrates how relational vulnerability, HIV‐related stigma, disclosure decisions and treatment‐related challenges intersect in the experiences of women living with HIV in a semiurban Indonesian setting. Participants′ accounts highlight the importance of considering family relationships and community responses alongside the physical and psychological demands of long‐term ART. Family acceptance and practical assistance were described as valuable sources of support, but the findings do not justify assuming that family involvement is universally safe or beneficial. The principal conceptual contribution is the proposed Relational Buffering Model of HIV Care Engagement, which positions relational safety, confidentiality and women’s autonomy as important considerations when examining the potential protective role of family support. The model remains provisional and requires testing across populations and healthcare settings. For nursing practice, the findings support individualised stigma‐informed counselling, voluntary family‐inclusive care when appropriate, and confidential alternatives for women who cannot safely disclose their HIV status. Future multisite, longitudinal and implementation studies should determine whether these approaches improve treatment engagement, remain acceptable and safe, and can be sustained within routine healthcare services. By connecting women’s lived experiences with testable theoretical propositions and a staged research agenda, this study provides a foundation for developing more relationally responsive and ethically grounded HIV care.

## Funding

This research was funded by Airlangga University for 2023 with No.: 809/UN.3.1.13/PT/2023.

## Disclosure

The funder was not involved in the research design, data collection, analysis, interpretation of results, preparation of the manuscript or publication decisions.

## Ethics Statement

Ethical approval was obtained from the Health Research Ethics Commission of the Faculty of Nursing and Health, Muhammadiyah University Semarang, Indonesia (No. 103/KE/07/2023). This ethics committee is authorised to review collaborative external human‐subject research.

## Conflicts of Interest

The authors declare no conflicts of interest.

## Supporting Information

Additional supporting information can be found online in the Supporting Information section.

## Supporting information


**Supporting Information** To safeguard participant privacy, the supporting information provided has been heavily sanitised. It includes a de‐identified thematic coding framework, the completed COREQ 32‐item checklist and a methodological audit trail. Raw demographic datasets, verbatim quotes and interview logs are intentionally excluded from public or supplementary sharing to prevent deductive disclosure within the local community, in strict accordance with the approved ethical protocols.

## Data Availability

The data supporting the findings of this study are not publicly available because the interview material concerns a sensitive health condition and disclosure could create a risk of participant re‐identification. However, to ensure methodological transparency, a sanitised Supporting File 1 has been provided, which includes a de‐identified thematic coding framework, the methodological audit trail and the COREQ checklist. Further de‐identified supporting materials may be made available by the corresponding author upon reasonable request, subject to ethical approval and strict safeguards for participant confidentiality.
