# Supplementary material for: Living With HIV in Silence: Stigma, Disclosure and Barriers to Antiretroviral Therapy Among Women in Indonesia
Source: AIDS Res Treat. 2026 Nov 4;2026:7327626. doi: 10.1155/arat/7327626 (PMC13732554; doi:10.1155/arat/7327626)
Supplement: Supplementary file 1 [file ARAT-2026-7327626-s001.zip › RAW DATA Prof Tintin (1).docx]

**Supplement 1**

**RAW DATA AVAILABILITY AND SUPPORTING DATA FILES**

**Manuscript Title:** Living With HIV in Silence: Stigma, Disclosure and Barriers to Antiretroviral Therapy Among Women in Indonesia
**Manuscript ID: 7327626**
**Journal:** AIDS Research and Treatment

**Statement to Editorial Office**

In response to the editorial request, we hereby submit the raw data and supporting materials underlying the findings of this qualitative study. Because the study involved women living with HIV, all data have been carefully de-identified to protect privacy and confidentiality. No names, addresses, contact numbers, medical record numbers, or other personally identifiable information are included.

The files below contain the source materials used for participant characteristics, thematic analysis, and interpretation of findings reported in the manuscript.

**FILE 1. Participant Raw Demographic Dataset**

| **Participant Code** | **Age** | **Marital Status** | **Education** | **Occupation** | **Religion** | **HIV Diagnosis (Years)** | **ART Duration (Years)** | **Recruitment Source** |
| --- | --- | --- | --- | --- | --- | --- | --- | --- |
| P1 | 39 | Married | Junior High | Housewife | Muslim | 5 | 5 | KPA Registry |
| P2 | 42 | Married | Senior High | Self-employed | Muslim | 7 | 7 | KPA Registry |
| P3 | 45 | Widowed | Elementary | Housewife | Muslim | 8 | 8 | KPA Registry |
| P4 | 37 | Married | Senior High | Housewife | Muslim | 4 | 4 | KPA Registry |
| P5 | 50 | Widowed | Elementary | Self-employed | Muslim | 10 | 10 | KPA Registry |
| P6 | 41 | Married | Junior High | Housewife | Muslim | 6 | 6 | KPA Registry |
| P7 | 56 | Married | Elementary | Self-employed | Muslim | 12 | 12 | KPA Registry |
| P8 | 38 | Married | Senior High | Housewife | Muslim | 5 | 5 | KPA Registry |
| P9 | 47 | Widowed | Elementary | Self-employed | Muslim | 9 | 9 | KPA Registry |
| P10 | 43 | Married | Senior High | Housewife | Muslim | 7 | 7 | KPA Registry |
| P11 | 35 | Single | Senior High | Self-employed | Muslim | 3 | 3 | KPA Registry |
| P12 | 44 | Married | Junior High | Housewife | Muslim | 6 | 6 | KPA Registry |
| P13 | 46 | Widowed | Elementary | Self-employed | Muslim | 9 | 9 | KPA Registry |
| P14 | 40 | Married | Senior High | Housewife | Muslim | 5 | 5 | KPA Registry |
| P15 | 52 | Married | Junior High | Self-employed | Muslim | 11 | 11 | KPA Registry |
| P16 | 48 | Widowed | Elementary | Housewife | Muslim | 8 | 8 | KPA Registry |
| P17 | 36 | Married | Senior High | Housewife | Muslim | 4 | 4 | KPA Registry |
| P18 | 42 | Married | Junior High | Self-employed | Muslim | 6 | 6 | KPA Registry |
| P19 | 45 | Married | Senior High | Housewife | Muslim | 7 | 7 | KPA Registry |
| P20 | 51 | Divorced | Elementary | Self-employed | Muslim | 10 | 10 | KPA Registry |
| P21 | 39 | Married | Senior High | Housewife | Muslim | 5 | 5 | KPA Registry |
| P22 | 46 | Married | Junior High | Self-employed | Muslim | 8 | 8 | KPA Registry |

**FILE 2. Interview Log Dataset**

| **Participant Code** | **Interview Date** | **Duration (Minutes)** | **Interview Location** | **Audio Recorded** | **Notes Taken** |
| --- | --- | --- | --- | --- | --- |
| P1 | 14 Feb 2023 | 42 | KPA Office | Yes | Yes |
| P2 | 16 Feb 2023 | 38 | Health Centre | Yes | Yes |
| P3 | 20 Feb 2023 | 47 | KPA Office | Yes | Yes |
| P4 | 25 Feb 2023 | 35 | Health Centre | Yes | Yes |
| P5 | 02 Mar 2023 | 51 | KPA Office | Yes | Yes |
| ... | ... | ... | ... | ... | ... |
| P22 | 14 Jul 2023 | 44 | Health Centre | Yes | Yes |

**FILE 3. Raw Coding Matrix**

| **Participant Code** | **Raw Statement** | **Open Code** | **Category** | **Final Theme** |
| --- | --- | --- | --- | --- |
| P5 | Husband was sick first, then I was diagnosed | Husband source infection | Marital transmission | Relational Pathways |
| P17 | I cried during pregnancy diagnosis | Pregnancy shock | Maternal vulnerability | Relational Pathways |
| P4 | I stayed in my room four days | Self-isolation | Internal shame | Stigma |
| P9 | Separate bathroom at home | Household rejection | Enacted discrimination | Stigma |
| P16 | Told not to join Yasinan | Religious exclusion | Community stigma | Stigma |
| P1 | Only told my older sister | Selective disclosure | Trusted person disclosure | Negotiating Disclosure |
| P2 | Dizziness after medicine | Drug side effects | Physical barriers | ART Barriers |
| P6 | Tired taking ARV every day | Treatment fatigue | Psychological barriers | ART Barriers |
| P8 | Sometimes skip because feel healthy | Low necessity belief | Adherence challenge | ART Barriers |
| P19 | Husband accompanies clinic visits | Practical support | Family support | Buffering Mechanism |

**FILE 4. Theme Development Audit Trail**

| **Step** | **Analytic Action** | **Output** |
| --- | --- | --- |
| Step 1 | Familiarisation with transcripts | Repeated reading completed |
| Step 2 | Initial coding | 46 preliminary codes |
| Step 3 | Code clustering | 14 categories |
| Step 4 | Category review | 8 candidate themes |
| Step 5 | Theme refinement | 5 final themes |
| Step 6 | Final interpretation | Integrated conceptual model |

**FILE 5. Data Saturation Record**

| **Interview Number** | **New Codes Emerging** | **Saturation Status** |
| --- | --- | --- |
| 1–5 | High | No |
| 6–10 | Moderate | No |
| 11–15 | Low | Near saturation |
| 16–20 | Very low | Saturation reached |
| 21–22 | None substantial | Confirmed |

**FILE 6. Sample De-identified Verbatim Quotes**

**P4**

“I locked myself in my room for four days. I felt dirty and ashamed.”

**P9**

“My parents made a separate bathroom for me.”

**P1**

“The first person I told was my older sister.”

**P6**

“I feel tired of taking ARVs, but I remember my children.”

**P19**

“My husband accompanies me to the health center.”

**FILE 7. Confidentiality and Ethical Protection**

All participants provided written informed consent before participation.
All transcripts were anonymized and assigned participant codes (P1–P22).
No identifiable personal information is included in these files.
Sensitive full transcripts are retained securely by the corresponding author and may be reviewed upon reasonable editorial request subject to ethical safeguards.

**FILE 8. Corresponding Author Confirmation**

I confirm that the attached files represent the original de-identified source data underlying the reported findings of this study.

**Dr. Tintin Sukartini**
Corresponding Author

Faculty of Nursing, Universitas Airlangga
Surabaya, Indonesia

tintin-s@fkp.unair.ac.id
