# Supplementary material for: Living With HIV in Silence: Stigma, Disclosure and Barriers to Antiretroviral Therapy Among Women in Indonesia
Source: AIDS Res Treat. 2026 Nov 4;2026:7327626. doi: 10.1155/arat/7327626 (PMC13732554; doi:10.1155/arat/7327626)
Supplement: Supplementary file 1 [file ARAT-2026-7327626-s001.zip › SUPPLEMENTARY FILE 1 prof tintin.docx]

# SUPPLEMENTARY FILE 1

## Methodological Rigor, Audit Trail, and Analytical Framework

**Manuscript Title:** Living With HIV in Silence: Stigma, Disclosure and Barriers to Antiretroviral Therapy Among Women in Indonesia

**Authors:** Tintin Sukartini, Nursalam, Erna Dwi Wahyuni, Rio Ady Erwansyah, Achmad Masfi, Ronal Surya Aditya

**Corresponding Author:** Tintin Sukartini (tintin-s@fkp.unair.ac.id)

## ETHICAL NOTE ON DATA SHARING

In strict accordance with the ethical approval granted by the Health Research Ethics Commission of the Faculty of Nursing and Health, Universitas Muhammadiyah Semarang (No.103/KE/07/2023), and to prevent deductive disclosure in a small, close-knit semi-urban community, this supplementary file has been heavily sanitised. Raw demographic datasets, verbatim interview transcripts, and identifiable interview logs are intentionally excluded from public or supplementary sharing. The materials provided below are designed to demonstrate analytical transparency and methodological rigor without compromising participant confidentiality.

## SECTION 1: COREQ 32-Item Checklist (Consolidated Summary)

This study adheres to the Consolidated Criteria for Reporting Qualitative Research (COREQ). Key domains are summarized below:

### Domain 1: Research Team and Reflexivity

- **Interviewer/Facilitator:** Two female researchers (TS, RSA) with backgrounds in mental health nursing and qualitative research training.
- **Credentials:** Nursing lecturers and researchers involved in HIV-related community health programs.
- **Relationship with Participants:** No prior relationship. Researchers explicitly clarified their role as independent researchers, not treating clinicians, to mitigate power dynamics.
- **Participant Characteristics:** Provided in Table 1 of the main manuscript (age, marital status, education, occupation, religion).
- **Setting:** Private, comfortable locations chosen by participants (KPA offices, community health facilities).
- **Repeat Interviews:** None conducted.
- **Data Saturation:** Explicitly discussed and confirmed at interview 20, with 2 additional interviews for verification (total n=22).

### Domain 2: Study Design

- **Theoretical Framework:** Constructivist principles informing a qualitative descriptive design.
- **Participant Selection:** Purposive sampling to ensure maximum variation in age, education, and duration of HIV infection.
- **Approach:** Semi-structured, in-depth interviews guided by a validated interview protocol.
- **Interview Guide:** Validated by a panel of experts in qualitative research and HIV clinical care prior to data collection.
- **Audio/Visual Recording:** Audio-recorded with explicit consent; field notes taken for non-verbal cues.
- **Field Notes:** Maintained to document contextual observations and non-verbal communication.
- **Duration:** Approximately 30–60 minutes per interview.
- **Transcripts Returned:** Not returned to participants for comment/correction, to avoid causing secondary psychological distress.

### Domain 3: Analysis and Findings

- **Number of Data Coders:** Two researchers (TS, RSA) coded independently.
- **Coding Tree:** Developed iteratively (see Section 4).
- **Derivation of Themes:** Inductive thematic analysis following Braun and Clarke's six-step framework.
- **Participant Checking:** Not formally conducted; however, analytic interpretations were reviewed collaboratively within the research team to ensure consistency with narratives.
- **Quotations Presented:** Yes, in the main manuscript (anonymized with participant codes, e.g., P1, P5).
- **Data and Findings Consistent:** Yes, themes are directly supported by anonymized participant narratives.
- **Clarity of Major Themes:** Yes, five interconnected themes are clearly presented.
- **Minor Themes:** Subthemes are detailed within the main thematic structure.

## SECTION 2: Aggregated Participant Characteristics Summary

*(Note: Detailed individual demographic data is withheld to prevent re-identification. Aggregate characteristics are reported in Table 1 of the main manuscript.)*

- **Total Participants:** 22 women living with HIV.
- **Age Range:** 35–56 years (Mean: 43 years).
- **Marital Status:** Married (n=15), Widowed (n=5), Divorced (n=1), Single (n=1).
- **Education:** Elementary (n=4), Junior High (n=5), Senior High (n=9), Higher Education (n=4).
- **Occupation:** Housewife (n=10), Self-employed (n=12).
- **Religion:** Muslim (n=21), Other (n=1).
- **Recruitment Source:** Local AIDS Prevention Commission (KPA) Registry.

## SECTION 3: Interview Protocol Summary

*(Note: Exact dates and specific locations are withheld to protect participant privacy.)*

- **Data Collection Period:** February – July 2023.
- **Interview Duration:** 30–60 minutes per participant.
- **Interview Locations:** Private rooms at KPA offices or community health centres, chosen by participants for comfort and privacy.
- **Method:** Semi-structured, in-depth interviews, audio-recorded with explicit consent, supplemented by researcher field notes.
- **Saturation:** Confirmed at interview 20, with 2 additional interviews conducted to verify no new thematic insights emerged.

## SECTION 4: De-identified Thematic Coding Framework

This table illustrates the analytical progression from initial inductive codes to final themes, demonstrating the rigor of the thematic analysis without exposing identifiable verbatim data.

| **Final Theme** | **Subtheme** | **Representative Initial Inductive Codes (Anonymized)** |
| --- | --- | --- |
| **1. Relational Pathways of HIV Transmission** | Discovery within marriage | "husband's prolonged illness", "shock during pregnancy", "betrayal vs. resignation" |
|  | Limited negotiation power | "financial dependence", "inconsistent condom use", "fear of asking" |
| **2. Internalised and Enacted Stigma** | Shame and self-isolation | "locked in room", "felt dirty", "fear of being labelled immoral", "hiding for years" |
|  | Community discrimination | "separate bathroom", "excluded from Yasinan", "gossip in the neighborhood" |
| **3. Negotiating Disclosure** | Selective trust | "told older sister first", "needed someone to understand", "testing the waters" |
|  | Fear of consequences | "blamed for the infection", "worried about remarriage prospects", "delayed telling spouse" |
| **4. Physical and Psychological Barriers to ART** | Medication side effects | "dizzy and nauseous", "anxiety about pills", "uncertainty due to low education" |
|  | Treatment fatigue & perceived wellness | "tired of taking ARVs", "feel fine so I skip", "cognitive dissonance about chronicity" |
| **5. Family Support as a Buffering Mechanism** | Practical and emotional reassurance | "sister reminds me", "husband accompanies to clinic", "tangible validation of worth" |
|  | Counteracting enacted stigma | "feeling accepted at home", "less isolated", "contrast with community rejection" |

## SECTION 5: Methodological Audit Trail

To ensure confirmability and dependability, the research team maintained a detailed audit trail documenting the analytical decision-making process:

1. **Initial Familiarization & Independent Coding:** Transcripts were read multiple times by TS and RSA. Initial codes were generated inductively in Bahasa Indonesia to preserve cultural nuance (e.g., retaining terms like *aib* and *malu* during initial coding).
2. **Translation & Conceptual Equivalence:** A forward-backward translation method was used. Discrepancies in translating culturally nuanced terms were resolved through team consensus to ensure the English manuscript accurately reflected the emotional weight of the original Bahasa Indonesia narratives.
3. **Collaborative Theme Development:** TS and RSA met bi-weekly to compare coding trees. Discrepancies (e.g., whether a code belonged to "Internalised Stigma" or "Negotiating Disclosure") were debated and resolved through consensus, referencing the original transcript context.
4. **Reflexive Bracketing:** Given the researchers' backgrounds as mental health nurses, reflexive journals were used to document and bracket clinical assumptions (e.g., the urge to "fix" non-adherence) to ensure the analysis remained grounded in the participants' lived experiences rather than clinical judgments.
5. **Final Review:** The broader research team (including authors from public health and community nursing backgrounds) reviewed the final thematic structure to challenge interpretive biases and ensure coherence.

## SECTION 6: Data Saturation Tracking Record

Saturation was monitored iteratively during data collection to determine the optimal sample size:

- **Interviews 1–10:** Rapid generation of new, diverse codes related to transmission shock, severe internalised stigma, and initial ART side effects.
- **Interviews 11–15:** Emergence of nuanced subthemes, particularly regarding the negotiation of disclosure with specific family members (e.g., siblings vs. spouses) and the concept of *aib* (family disgrace).
- **Interviews 16–20:** Code frequency stabilized. No new major themes emerged. Existing themes were richly populated with confirming and slightly varying instances (e.g., variations in how family support was experienced).
- **Interviews 21–22:** Conducted explicitly to confirm theoretical saturation. Analysis of these two interviews yielded **zero new codes or conceptual insights**. The existing five themes comprehensively captured the phenomenon. Recruitment was subsequently halted.

## SECTION 7: Confidentiality and Ethical Protection Protocol

1. All participants provided written informed consent before participation.
2. All transcripts were immediately anonymized and assigned generic participant codes (e.g., P1–P22).
3. No identifiable personal information (names, exact addresses, specific dates, or unique demographic combinations) is included in this supplementary file or the main manuscript.
4. Sensitive full transcripts and raw audio recordings are retained securely in a password-protected drive accessible only to the core research team, in strict compliance with institutional ethical guidelines.

## SECTION 8: Statement on the Absence of Raw Data

**Reviewer and reader transparency note:**

While some qualitative research paradigms encourage the sharing of raw transcripts, this study deliberately withholds them. The participants reside in a semi-urban regency (Tulungagung) with a highly interconnected social fabric. The combination of specific demographic variables (e.g., age, marital status, occupation), unique life events (e.g., specific timing of diagnosis during pregnancy), and verbatim speech patterns creates a high risk of **deductive disclosure**, even if names are removed.

Therefore, to uphold the highest ethical standard of *non-maleficence* and to honor the informed consent agreements which guaranteed absolute confidentiality, this supplementary file provides the analytical framework and audit trail as proof of rigor, rather than raw data. De-identified thematic structures and methodological logs are available upon reasonable request to the corresponding author, subject to institutional ethical review.

## SECTION 9: Corresponding Author Confirmation

I confirm that the attached sanitised files represent the analytical framework and methodological audit trail underlying the reported findings of this study. Raw data is withheld solely to protect participant confidentiality, as approved by our ethics committee.

**Dr. Tintin Sukartini**
Corresponding Author
Faculty of Nursing, Universitas Airlangga, Surabaya, Indonesia
Email: tintin-s@fkp.unair.ac.id
